# A nursing‐led pathway to safe task sharing in Japan: Interprofessional simulation and a common competency credential

**DOI:** 10.1111/jjns.70036

**Published:** 2025-12-08

**Authors:** Kazumi Kubota, Ayako Nishimura, Jun Funaki

**Affiliations:** ^1^ Research Organization Shimonoseki City University Yamaguchi Japan; ^2^ Department of Healthcare Information Management The University of Tokyo Hospital Tokyo Japan; ^3^ Health and Global Policy Institute Tokyo Japan; ^4^ Department of Biostatistics, Graduate School of Medicine Yokohama City University Yokohama Japan; ^5^ Faculty of Healthcare, Division of Nursing Tokyo Healthcare University Tokyo Japan; ^6^ Major in Nursing Science, Medical School Department of Health Science, Graduate School of Nursing Nagoya City University Nagoya Japan

**Keywords:** competency‐based education, credentialing, interprofessional simulation, Japan, nursing leadership, task sharing

## Abstract

**Aim:**

To propose a nursing‐led pathway for safe task sharing in Japan to address population aging and workforce shortages.

**Methods:**

This Viewpoint outlines a practical route centering on five shared competency domains (decision‐making, safety management, communication, equipment operation, and troubleshooting) verified through interprofessional simulation. We describe a common competency credential (CCC) assessed via standardized scenarios, objective rating scales, and mastery thresholds.

**Results:**

A nursing‐led policy pathway would align education, credentialing, and financing via the national fee schedule. By embedding stop rules, clear supervision levels, and psychological safety, the approach addresses concerns regarding safety, legal accountability, and hierarchy in the Japanese context.

**Conclusions:**

With the 2024 fee schedule emphasizing team‐based care, nursing‐led interprofessional simulation and a CCC offer a feasible, evidence‐informed route to safer care, greater efficiency, and a more sustainable workforce.

Japan continues to grapple with rapid population aging, rising multimorbidity, and persistent workforce shortages. Many health systems have responded to similar pressures by sharing tasks: moving well‐defined clinical activities from physicians to other professionals under protocols and supervision. A substantial body of evidence shows that when teams are trained and oversight is clear, outcomes for selected tasks remain comparable to physician‐led care, while access and efficiency improve (Laurant et al., 2018; Maier & Aiken, 2016; World Health Organization [WHO], 2008). In Japan, the trajectory has been cautious. The 2015 “specified acts” framework for nurses marked progress, yet broader engagement of allied health professionals remains limited (Ministry of Health, Labour and Welfare [MHLW], 2014; Organisation for Economic Co‐operation and Development [OECD], 2016). With physician work‐hour caps now enforced and the 2024 fee schedule emphasizing team‐based care, there is a timely opportunity to scale safe task sharing (MHLW, 2024).

This Viewpoint argues that nursing can lead an evidence‐informed pathway by focusing on common, transferable competencies across professions and by credentialing those competencies through interprofessional simulation. The approach fits Japan's regulatory context and addresses recurring concerns regarding safety, liability, and hierarchy. Nurses constitute the largest and most continuously present professional group at the point of care. They already practice within the specified acts system and benefit from established educational and quality governance structures. Nursing has also adopted simulation‐based education and assessment at scale, with evidence that high‐quality simulation can replace a meaningful share of clinical hours without compromising competence (Hayden et al., 2014). These capacities position nursing to convene clinical engineers, clinical laboratory technologists, and others around a shared competency agenda and to model rigorous training and assessment for task sharing.

A common competency core underpins safe redistribution of tasks.

Five domains are central: task‐level clinical decision‐making, technical safety management, interprofessional communication, equipment operation, and troubleshooting. Task‐level decision‐making entails recognizing salient cues, applying protocols, and escalating appropriately. Technical safety management includes sterile technique, device and medication safety checks, and the use of fallbacks and stop rules. Communication relies on shared frameworks such as SBAR and closed‐loop confirmation to support clarity and accountability (Haig et al., 2006). Equipment operation involves correct setup, parameter verification, and basic maintenance for common devices. Troubleshooting draws on systematic fault finding and recovery, combined with the judgment to pause and request assistance when thresholds are reached.

Technology‐enhanced simulation improves knowledge, procedural skills, teamwork behaviors, and transfer to practice (Cook et al., 2011; Issenberg et al., 2005; McGaghie et al., 2014; Weaver et al., 2014). Interprofessional designs strengthen role clarity, shared mental models, and coordination—capabilities that support safe task sharing (Reeves et al., 2016; Salas et al., 2008). Debriefing using established approaches, such as the PEARLS framework, consolidates learning and supports behavior change, while psychological safety enables teams to surface latent threats and learn from error (Edmondson, 1999; Eppich & Cheng, 2015). These features are mapped closely to the five competency domains and offer practical routes to measurable proficiency.

Building on this evidence, we propose a common competency credential (CCC) that verifies proficiency in the five domains through standardized, simulation‐based assessment. The CCC would be profession‐agnostic yet context‐sensitive. Nurses, clinical engineers, and laboratory technologists would complete role‐relevant scenarios but be judged against the same domain standards. Governance would be shared by nursing organizations, allied health associations, and national bodies, integrating educational, regulatory, and practice expertise. The assessment system would draw on a standardized scenario library comprising high‐fidelity cases from ward, intensive care, and diagnostic workflows. Each case would embed explicit supervision levels, stop rules, checklists, and escalation criteria to reflect real‐world safety boundaries. Objective assessment would employ validated teamwork and technical scales and behaviorally anchored checklists, with trained raters and interrater reliability audits to support fairness and reproducibility (Mitchell & Ivimey‐Cook, 2023; Weaver et al., 2014). Proficiency thresholds would be set by expert panels, and re‐credentialing would occur every 3–5 years or after significant scope changes, with remediation pathways for those not meeting the standard (McGaghie et al., 2014). Documentation would delineate delegated tasks, supervision levels, and escalation triggers in alignment with institutional policies and Japanese law, clarifying responsibility and liability.

A nursing‐led policy pathway can activate the CCC through four complementary levers: education, credentialing and quality assurance, financing and incentives, and measurement within a learning system. In education, interprofessional simulation modules should be integrated into prelicensure and continuing programs, substituting part of clinical hours where quality standards are met (Hayden et al., 2014). Faculty capacity in scenario design and debriefing warrants investment, with emphasis on psychological safety and advocacy‐inquiry techniques that promote reflection and shared understanding (Edmondson, 1999; Eppich & Cheng, 2015). Shared cases can normalize cross‐professional perspective taking and reduce hierarchical barriers that slow escalation.

Figure 1 summarizes how these elements fit together—from the competency core, through education and assessment, to policy levers and outcomes—so that readers can see the overall logic before turning to governance and assurance. In credentialing and quality assurance, a nursing‐led governance board could launch the CCC, progressively adding allied health representation and external examiners to ensure legitimacy and breadth. Rater training and reliability checks would be standard, with a national registry of credentialed practitioners and accredited simulation centers to support transparency and mobility. Institutional policies should recognize the scope associated with the CCC within clear supervision frameworks, ensuring that the credential translates into safe task sharing in daily care.

**FIGURE 1:**
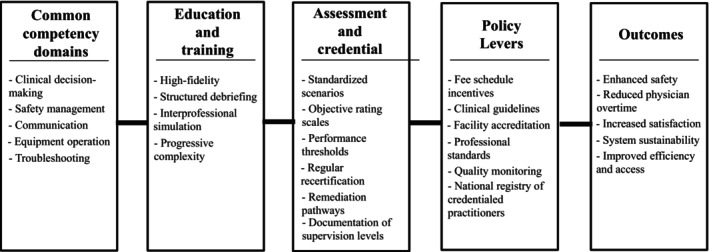
Nursing‐led pathway to safe task sharing in Japan. Common competency domains feed into interprofessional simulation and standardized assessment to support a national common competency credential (CCC), activated by education, financing, and quality assurance levers, and leading to improvements in safety, efficiency and access, clinician workload, satisfaction, and system sustainability.

Financing and incentives are essential for adoption. The national fee schedule can recognize training costs and the use of credentialed staff in designated services, following precedents where reimbursement accelerated team‐based care (MHLW, 2024). Time‐limited add‐on payments could support early adopters and catalyze diffusion in priority areas, particularly where physician overtime is high or access gaps are pronounced. Aligning incentives with safety and quality standards helps ensure that scaling does not outpace capability.

Measurement within a learning system links the initiative to outcomes that matter. Process indicators, such as time to intervention and adherence to safety checks, can be paired with outcome measures, including adverse events, throughput, and patient experience. These metrics should appear on institutional dashboards to enable continuous improvement. Pragmatic trials and implementation studies can evaluate impact and cost effectiveness in routine settings (Cook et al., 2011; Laurant et al., 2018). National collaboratives and simulation competitions can accelerate shared learning across regions and build a community of practice that sustains momentum (Reeves et al., 2016).

Safety, legal liability, and culture merit explicit attention. Safety is the foremost criterion. The CCC embeds stop rules, defined supervision levels, and scripted escalation to manage risk at the point of care. Standardization reduces unwarranted variation, and simulation provides a safe environment to identify and remedy system defects before they reach patients (Issenberg et al., 2005; McGaghie et al., 2014). Institutions should align CCC scopes with bylaws, credentialing committees, and risk management processes, with documentation and clear lines of responsibility to mitigate medicolegal ambiguity. Cultural barriers in hierarchical settings can be moderated by fostering psychological safety and using structured communication tools such as SBAR, which encourage timely voice across professional boundaries (Edmondson, 1999; Haig et al., 2006).

The timing in Japan is favorable. The 2024 fee schedule emphasizes team‐based care as a strategy to curb physician overtime and sustain quality under demographic and fiscal pressure (MHLW, 2024). The nursing “specified acts” infrastructure offers a tested training model, and simulation capacity has expanded across nursing schools and tertiary centers. By starting with common competencies rather than professional titles, Japan can scale capability more quickly, respect professional identities, and make task sharing a property of well‐prepared teams.

A focused research and evaluation agenda should accompany implementation. Priorities include linking CCC adoption to clinical outcomes, near misses, and patient‐reported measures; quantifying training costs relative to productivity gains and avoided adverse events; assessing regional scaling and equity, including rural, community, and home care settings; estimating time to proficiency across domains and professions and optimizing debriefing strategies; and examining how fee schedule incentives and institutional policies influence uptake and sustainability. These studies can strengthen the causal case for the CCC and inform iterative refinement of standards and scenarios.

Task sharing in Japan will advance if it is safe, measurable, and workable in local culture. Nursing can lead by concentrating on common competencies and by credentialing them through high‐quality interprofessional simulation. A common competency credential, aligned with education, financing, and measurement, provides a feasible route to safer care, better team efficiency, and a more sustainable workforce—turning team‐based care from aspiration into routine practice.

## AUTHOR CONTRIBUTIONS

*Conceptualization and methodology*: Ayako Nishimura, Kazumi Kubota, and Jun Funaki. *Data collection*: Kazumi Kubota and Ayako Nishimura. *Data curation*: Kazumi Kubota. *Project administration*: Kazumi Kubota. *Resources*: Kazumi Kubota, Ayako Nishimura, and Jun Funaki. *Manuscript drafting*: Kazumi Kubota. *Critical revision of the manuscript for important intellectual content*: Ayako Nishimura and Jun Funaki.

## CONFLICT OF INTEREST STATEMENT

The authors declare that there is no conflict of interest.
